# Mortality Prediction Among People Living With HIV on Antiretroviral Therapy in Public Health Facilities in Gondar City Administration, Northwest Ethiopia: Machine Learning–Based Study

**DOI:** 10.2196/78770

**Published:** 2026-04-20

**Authors:** Andualem Enyew Gedefaw, Getaye Tizazu Biwota, Abraraw Gebre Mesele, Abraham Keffale Mengistu, Gizaw Hailiye Teferi

**Affiliations:** 1 Department of Health Informatics, Institute of Public Health, College of Medicine and Health Sciences, University of Gondar, P.O. Box 196, Hospita, Gondar, Ethiopia, 251 918356802; 2 Department of Health Informatics, College of Medicine and Health Sciences, Debre Markos University, Debre Markos, Ethiopia

**Keywords:** HIV mortality prediction, machine learning, ensemble algorithms, Shapley Additive Explanations, SHAP analysis, antiretroviral therapy, baseline predictors, electronic medical records

## Abstract

**Background:**

Predicting mortality among people living with HIV enables clinicians to implement timely, targeted, and preventive interventions at the start of antiretroviral therapy (ART). However, prognostic models must rely strictly on baseline predictors to avoid look-ahead bias and ensure scientific validity. This study evaluates machine-learning (ML) algorithms for baseline mortality prediction using routine electronic medical record data.

**Objective:**

This study aims to predict mortality among people living with HIV receiving ART using baseline clinical and sociodemographic characteristics through ML models in public health facilities of Gondar City Administration, Northwest Ethiopia.

**Methods:**

The retrospective cohort study was conducted using electronic medical record data from 12,871 people living with HIV on ART (2005‐2024). Seven base classifiers were evaluated using stratified 10-fold cross-validation. Synthetic minority oversampling technique (SMOTE)–balanced variants were used only for sensitivity analysis. SMOTE oversampling was applied only to training folds; the final evaluation used the original imbalanced test data. Shapley Additive Explanations (SHAP) analysis identified key baseline predictors.

**Results:**

Gradient boosting on the original data achieved superior performance (accuracy 87.0%, *F*_1_-score 0.619, area under the receiver operating characteristic curve 0.859), outperforming extreme gradient boosting (*F*_1_-score 0.609, area under the receiver operating characteristic curve 0.835) and SMOTE variants. The SHAP analysis identified education level, lack of formal education (+0.84), and a low baseline cluster of differentiation 4 (CD4; a type of immune cell count) count of 140 cells/mm³ (+0.54) as substantially increasing predicted mortality risk. Urban residence (−0.35) and working functional status (−0.12) showed protective effects, whereas age (45 y; −0.02) had minimal influence in the illustrated case. Globally, lower CD4 counts and the absence of formal education were consistently associated with increased mortality risk.

**Conclusions:**

Ensemble ML models demonstrated moderate-to-strong discrimination for predicting mortality among people living with HIV using strictly baseline routine electronic medical record data. SHAP-based interpretability confirmed that educational attainment and baseline CD4 count were the dominant determinants of predicted mortality risk, underscoring the combined influence of socioeconomic vulnerability and immunological status at ART initiation. These findings support the potential utility of interpretable ML models for early risk stratification and targeted clinical decision-making in resource-limited settings; however, external validation is required before routine clinical implementation.

## Introduction

Recent advances in machine learning (ML) have enabled the development of predictive models using routinely collected electronic medical record (EMR) data to support clinical decision-making and risk stratification. Several studies demonstrated the feasibility of applying ML algorithms, such as logistic regression, random forest, gradient boosting, and extreme gradient boosting (XGBoost), to predict mortality and other adverse outcomes across diverse clinical settings using EMR or electronic health record data. Systematic reviews highlighted that ML-based mortality prediction models often outperform traditional statistical approaches, while also emphasizing the importance of appropriate validation strategies and the prevention of data leakage through baseline-only predictors [1-3]. EMR-based ML models have been successfully applied to predict short-term and in-hospital mortality among patients with conditions, such as cancer, heart failure, and COVID-19, using structured clinical and laboratory variables [4-6]. These studies underscore the growing role of explainable artificial intelligence (XAI) methods, such as SHAP (Shapley Additive Explanations), in improving the interpretability and clinical relevance of complex ML models, which is a key consideration for adoption in real-world health care settings [5,7].

HIV remains a major public health concern globally and in sub-Saharan Africa, where mortality among people living with HIV persists despite widespread ART coverage. Since the early 2000s, when the global HIV epidemic peaked, new infections and HIV-related deaths have declined. Expanded antiretroviral therapy (ART) coverage has reduced HIV-related mortality by almost 47%, from 2.1 million in 2004 to 630,000 in 2023 [8]. However, the epidemic remains a significant global health issue, especially in regions with limited access to preventive, diagnostic, and treatment services. Currently, an estimated 39.9 million individuals are living with HIV, according to the UNAIDS (Joint United Nations Program on HIV/AIDS) 2024 Global HIV and AIDS Statistics Report.

Sub-Saharan Africa bears the highest global HIV burden, accounting for approximately 67% of HIV cases and over 60% of HIV-related deaths [8]. Southern and Eastern African nations have made progress in reducing mortality and increasing access to ART. However, challenges, such as poverty, stigma, violence, and health care inequality, hinder efforts to decrease HIV-related fatalities. In 2023, approximately 390,000 Africans died from HIV-related causes. While ART rollouts have significantly reduced these numbers, the disease remains a serious public health concern.

Ethiopia has one of the largest HIV epidemics in East Africa, with approximately 610,000 people living with HIV in 2023 [2]. Despite improvements in health care infrastructure and ART coverage, significant challenges persist, especially for individuals with advanced HIV or treatment failure. In 2023, Ethiopia recorded approximately 10,000 HIV-related deaths, with rural areas facing higher mortality due to limited health care access [9]. HIV-related mortality remains high in sub-Saharan Africa, including Ethiopia, even though ART has transformed HIV into a chronic, manageable condition. Key contributors include late ART initiation, poor adherence, medication resistance, and virological failure, which prevent effective viral suppression [10-12]. Virological failure accelerates disease progression and increases the risk of AIDS-related death. Reducing mortality among people living with HIV requires early diagnosis, improved ART regimens, and better patient monitoring.

A 2022 study at the University of Gondar Comprehensive and Specialized Hospital reported a virological failure rate of 14% among people living with HIV on ART [12]. This highlights the urgent need for predictive tools to identify high-risk patients early and improve clinical outcomes. Mortality prediction plays a crucial role in health care, epidemiology, insurance, and policymaking. It helps health care providers identify high-risk individuals, enabling early interventions and personalized treatments that improve patient outcomes [13]. It also supports epidemiological research by analyzing disease patterns and assessing intervention effectiveness [8. Advanced methodologies, such as statistical models, ML, and deep learning, have significantly improved the accuracy and applicability of mortality predictions [14,15].

ML offers a data-driven solution for predicting mortality risk in people living with HIV on ART. ML algorithms analyze large datasets to detect complex patterns, risk factors, and interactions that traditional statistical methods may overlook [16]. This study develops interpretable ML models using strictly baseline predictors (recorded at ART initiation) from 12,871 people living with HIV EMRs to enable prospective mortality risk stratification. There is a pressing need to implement advanced predictive tools, such as ML models, to identify individuals at greater risk of treatment failure or death [17]. These tools can enable health care providers to take proactive measures, improve treatment outcomes, and reduce HIV-related mortality in Ethiopia.

This study aimed to develop and validate ensemble ML models predicting mortality among people living with HIV on ART in Gondar, Ethiopia, using baseline-only EMR data, with the SHAP analysis to identify clinically actionable risk factors. Several studies in Africa and Asia have applied ML for HIV-related outcomes, including mortality and treatment interruption. A meta-analysis across 24 studies found that ML models achieved a C-index of ~0.83 for HIV mortality prediction [18]. In China, an XGBoost and random forest ensemble achieved an area under the receiver operating characteristic (ROC) curve (AUC) of ~0.98 for in-hospital mortality among patients with HIV/AIDS with cryptococcal infection [19]. In Nigeria, a large EMR-based ML study of 41,394 people living with HIV reported an AUC of ~0.8 for treatment interruption [20]. However, these studies often used hospital-based cohorts or follow-up predictors, focused on treatment interruption or virological failure rather than long-term mortality, and lacked comprehensive interpretability analyses. Our study addresses these gaps through large-scale public health facility EMR data (n=12,871), strict baseline-only prediction, rigorous temporal validation, and SHAP-based clinical interpretability.

Traditional methods of monitoring and predicting patient outcomes rely on periodic clinical assessments, laboratory tests, and physician judgment. These approaches often detect complications only after symptoms have worsened, leading to delayed interventions that may not prevent adverse outcomes. Additionally, reliance on manual record keeping and retrospective data analysis limits the ability to identify high-risk patients early, reducing the effectiveness of timely medical interventions. In resource-constrained settings, such as the Gondar City Administration, integrating ML models could improve health care delivery, optimize resource distribution, and enhance proactive patient management [21]. This approach aligns with Ethiopia’s national HIV/AIDS control strategy and global health goals aimed at reducing HIV-related mortality.

## Methods

### Study Area and Period

Data were extracted from the ART EMRs covering the period from 2005 to 2024. Only patients on ART for ≥6 months before analysis were included to ensure baseline predictor availability.

This study was conducted at public health facilities in Gondar City Administration (Amhara Region, Ethiopia) from October 10 to December 10, 2024. Gondar City (population 457,938) is located 748 km northwest of Addis Ababa. In the Gondar City Administration, several health care facilities provide ART services to people living with HIV. Among them, the University of Gondar Specialized and Comprehensive Hospital serves as a key provider of ART services, offering specialized care to patients. Additionally, multiple health centers, including Azezo Health Center, Gondar Health Center, Maraki Health Center, Mintwab Health Center, St. Gebriel Health Center, Teda Health Center, and Woleka Health Center, also provide ART services, ensuring accessibility to treatment at various levels of health care.

**Figure 1.**
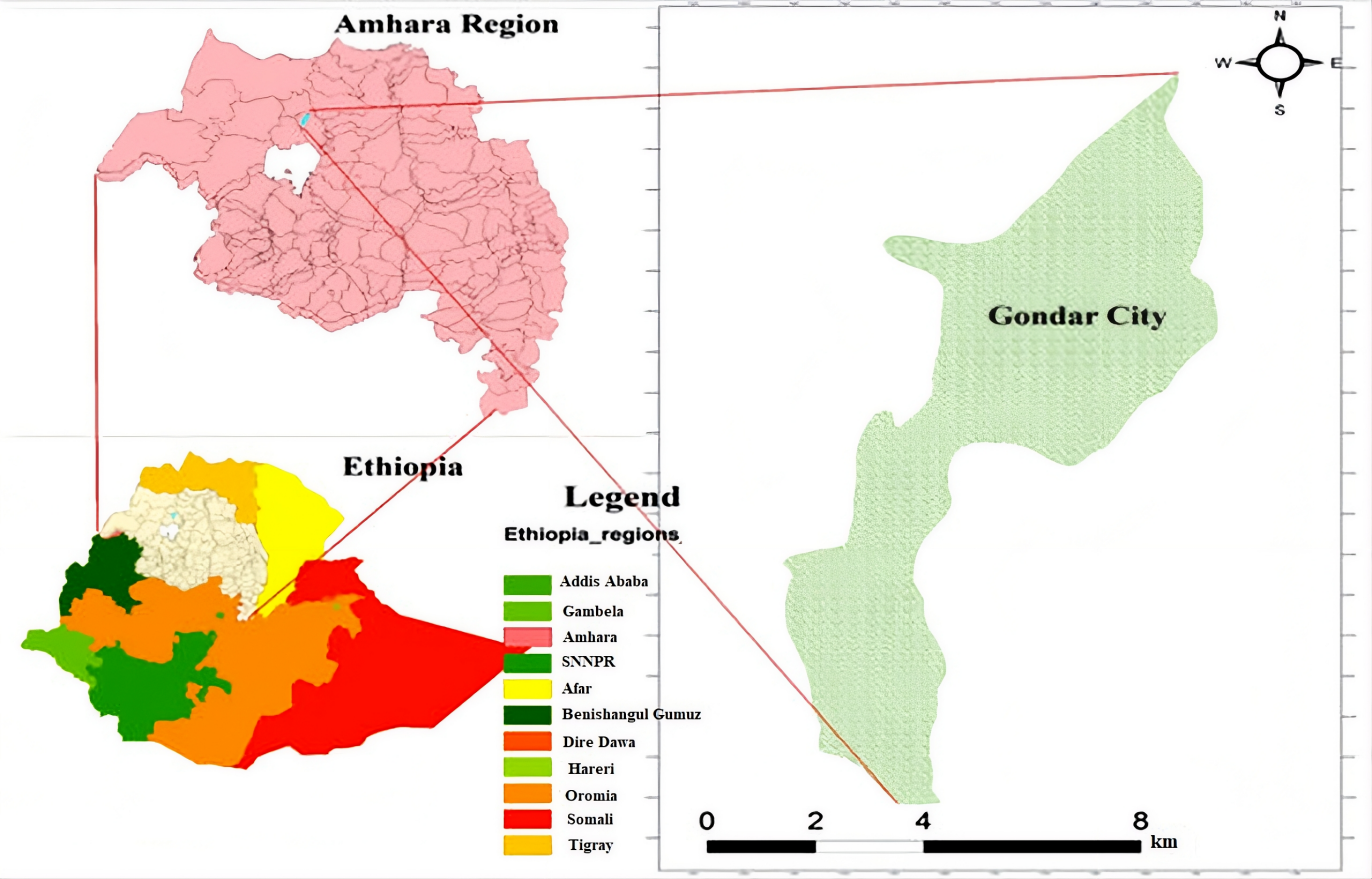
Geographic location of the study area showing the Gondar City Administration, Amhara National Regional State, Northwest Ethiopia, where a retrospective cohort study of adults living with HIV receiving antiretroviral therapy (ART) was conducted using electronic medical records from public health facilities, 2005‐2024. The map of the study area was adopted from Tamiru AT, Rade BK et al. (2020) [22].

Health facility mapping was conducted to provide context to the data source; however, facility service gaps were not the focus of this predictive modeling study (see Figure 1).

### Study Design

**Figure 2.**
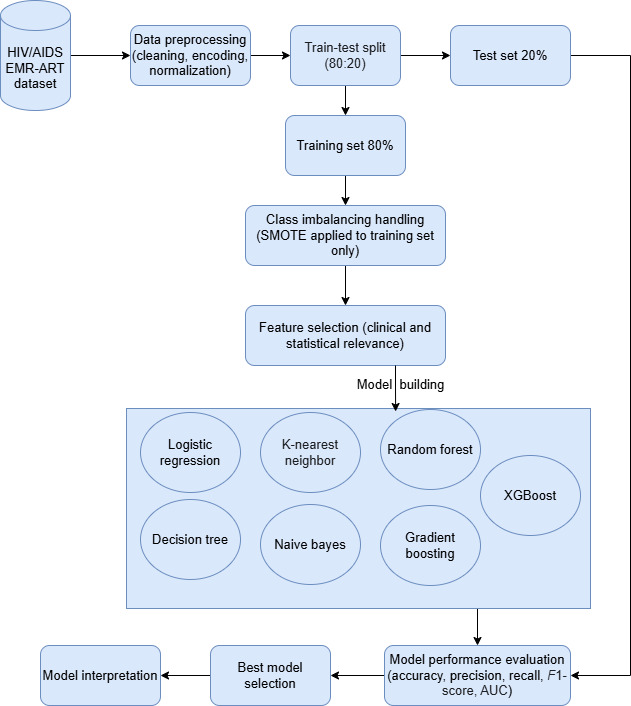
Workflow diagram of the machine-learning pipeline used to predict mortality among adults living with HIV on antiretroviral therapy (ART) in public health facilities of Gondar City Administration, Northwest Ethiopia, 2024. AUC: area under the receiver operating characteristic curve; EMR: electronic medical record; SMOTE: synthetic minority oversampling technique; XGBoost: extreme gradient boosting.

The retrospective cohort study analyzed baseline-only EMR data (ART initiation records) to predict prospective mortality (see Figure 2).

### Data Source

Data are sourced from EMR-ART databases across Gondar City Administration public health facilities, containing baseline sociodemographic, clinical, laboratory, and treatment initiation data.

#### Description of the Dataset

The dataset used in this study focused on people living with HIV undergoing ART in public health facilities within the Gondar City Administration, Ethiopia. Data collection covered the entire period since the start of ART services at the facilities.

#### Inclusion and Exclusion Criteria

Eligible participants were adults aged ≥18 years of age with ≥6 months of ART exposure. Participants were included if baseline variables were recorded at ART initiation; limited missing baseline values (<10%) were addressed using imputation. We excluded patients with incomplete ART start dates, those transferred to other facilities without outcome documentation, and non-HIV–related deaths (eg, accidents and trauma) to ensure outcome validity.

#### Predictor Variables

The binary outcome was mortality (follow-up status: alive=0, dead=1). Baseline predictors (all recorded at ART initiation) included 10 variables across 3 domains: sociodemographic (age, sex, marital status, educational level, residence, and religion), clinical (tuberculosis [TB] screening result and functional status), and immunological (baseline cluster of differentiation 4 [CD4] count). The baseline CD4 count was modeled as a continuous numerical feature in all ML algorithms. Descriptive statistics are therefore presented as median and IQR. Variable selection used recursive feature elimination with cross-validation applied exclusively to training data. The subsequent SHAP analysis confirmed the clinical plausibility of retained predictors.

#### Operational Definitions

Mortality, the outcome variable of interest in the study, is defined as the death of people living with HIV receiving ART. This is directly or indirectly related to the progression of HIV, opportunistic infections, or complications arising from the virus or treatment.

A low CD4 count of less than 200 cells/mm³ indicates a severely weakened immune system, placing the individual at high risk for opportunistic infections and HIV-related complications. A normal CD4 count between 200 and 500 cells/mm³ suggests a moderately functioning immune system. A high CD4 count greater than 500 cells/mm³ is considered within the normal range and reflects a healthier immune system.

Comorbidities are the presence of other chronic health conditions in people living with HIV alongside HIV infection.

#### Data Collection Tool and Procedures

The data collection tool used in this study was an electronic data extraction form specifically designed to retrieve relevant patient information from the EMR-ART database. The form was developed via a standardized template based on key study variables to ensure data consistency, completeness, and accuracy. The data collection process began with obtaining ethical approval and permission from relevant health authorities. Data collectors were trained in using the extraction tool and procedures to ensure uniformity in data retrieval. Data were extracted by reviewing patient records from the EMR-ART database using selected features and exported into Excel. Each record was thoroughly reviewed for accuracy, and a second data collector cross-verified entries to maintain data consistency.

### Ethical Considerations

Ethical approval for this study was obtained from the College of Medicine and Health Sciences Institutional Research Ethics Review Committee (CMHS IRERC) at Debre Markos University (reference number RCSTTD/403/01/17). Due to the retrospective design, the requirement for informed consent was waived by the committee in accordance with national research ethics guidelines.

All data used were deidentified before analysis to ensure participant privacy and confidentiality. No personally identifiable information (eg, names or medical record numbers) was extracted. Data were stored on password-protected computers accessible only to authorized researchers.

There was no financial compensation to participants, as this study relied on existing EMRs. No images or identifiable information about individual participants are presented in this paper. Ethical approval was obtained from the institutional review board of the Amhara Public Health Institute (APHI) (approval number: APHI/322/007). The study was conducted in accordance with the ethical principles of the Declaration of Helsinki and complied with the Ethiopian National Research Ethics Review Guideline [23].

### Data Quality Control

Data quality control procedures were applied throughout the study to ensure reliability and validity. These procedures involved consistency checks and the correction of missing entries. Data entry was supervised by experienced clerks, and predefined validation rules flagged inconsistencies.

### Data Management and Analysis

#### Association Rule Mining

Association rule mining was conducted to identify frequent co-occurrence patterns among clinical and treatment-related factors associated with mortality. The Apriori algorithm implemented in the mlxtend Python library was used. The mlxtend Python library was developed by Sebastian Raschka and provides useful extensions for machine learning and data science tasks. Key metrics included support (frequency of rule occurrence), confidence (conditional probability of the consequent given the antecedent), and lift (degree of dependence between antecedent and consequent). Rules with support ≥0.065, confidence ≥0.64, and lift >4.6 were retained for interpretation. These analyses were used to explore unadjusted co-occurrence patterns and were not intended to estimate causal or adjusted effects.

#### Data Cleaning

This phase involved handling missing values, detecting and removing outliers via Excel filters, and addressing class imbalance. The dataset contained less than 10% missing values for most variables. Median imputation was applied to numerical variables, such as CD4 count, while mode imputation was used for categorical baseline variables, such as marital status, residence, and religion. These simple imputation methods were chosen due to the relatively small proportion of missingness and to maintain data interpretability whereas mode imputation was applied to categorical data. One-hot encoding transforms categorical variables, creating separate columns for each category. To handle class imbalance, various techniques have been tested, with SMOTE (synthetic minority oversampling technique) providing the best accuracy. Other balancing techniques, such as random undersampling and cost-sensitive learning, were initially tested. However, they resulted in reduced sensitivity for the minority class (deceased patients). SMOTE provided the best balance between precision and recall, ensuring robust generalization across folds.

#### Feature Engineering

By converting raw data into meaningful features, feature engineering enhances model performance. One-hot encoding was used for categorical variables, and normalization ensured comparable feature scales [24].

#### Feature Selection and Dimensionality Reduction

Feature selection was performed exclusively using recursive feature elimination with cross-validation on the training data to identify the most informative subset of predictors while minimizing redundancy and overfitting.

#### Data Splitting

A training-test split and K-fold cross-validation ensured robust evaluation. The dataset was divided into training and testing sets, with K-fold validation reducing overfitting and improving generalizability.

#### Model Training and Evaluation

To prevent data leakage, the dataset was first split into training and hold-out test sets. All preprocessing steps, including imputation, feature scaling, encoding, and feature selection, were conducted exclusively within the training data using a cross-validation framework. Model performance was assessed using stratified 10-fold cross-validation on the training set and subsequently evaluated on the original, imbalanced hold-out test set. Sensitivity analyses were conducted by applying SMOTE to the training data only to assess the robustness of model performance under class imbalance.

#### Data Leakage Assessment and Stress-Test Validation

To ensure the robustness and validity of the predictive modeling framework, a series of predefined stress tests were conducted to assess potential data leakage, proxy outcome variables, and look-ahead bias.

#### Stress Test: Broken Split Check (Demographic-Only Model)

To verify independence between training and testing datasets, an XGBoost model was trained using only demographic variables (age and sex). The resulting model achieved an AUC of 0.631 on the hold-out test dataset, consistent with expected performance for demographic-only mortality prediction models. This finding confirmed that the data split procedure did not introduce artificial performance inflation due to overlapping observations or improper sampling procedures.

#### Model Selection

After the model was trained, several classifiers were evaluated to identify the most suitable model for predicting mortality among people living with HIV users. Given that the outcome variable is categorical and falls into 2 mutually exclusive groups, the problem was framed as a binary classification task. To select the best model, we compared the performance of these classifiers via evaluation metrics, such as accuracy, precision, recall, *F*_1_-score, and AUC [25]. During cross-validation, we examined the consistency of each model’s performance across different subsets of the data. The model with the best trade-off between predictive power and generalization to unseen data was chosen for final model selection. This approach ensured that the selected model was the right model for accurately predicting mortality among people living with HIV.

#### Evaluation Criteria

In this study, the performance of the predictive models was evaluated by testing a dataset within a training-test split and cross-validation. The performance of the trained models was subsequently evaluated on the test set based on the criteria of accuracy score, ROC curve, precision, recall, and *F*-measure. The confusion matrix, which is a matrix of N×N, where N is the number of predicted classes, displaying the number of correct and incorrect predictions made by the classification model, was used in this study. Because mortality prevalence was 20%, *F*_1_-score and AUC were prioritized over accuracy for model comparison.

## Results

### Description of Sociodemographic Characteristics

A total of 12,871 study participants were included in this study; the majority were between the ages of 38 and 47 years (n=4468, 34.7%), suggesting a largely middle-aged population; 7688 (59.7%) of the sample were female, indicating a gender imbalance; the majority were married (n= 4921, 51.2%) and had completed secondary school education (n=2953, 31.3%), indicating a moderate level of education; a sizable portion lived in urban areas (n=8878, 77.1%), suggesting greater representation of urban areas; and the vast majority were Orthodox Christians (n=8714, 91.5%), representing a highly homogeneous religious composition (see Table 1).

**Table 1.** Sociodemographic characteristics of adult people living with HIV receiving antiretroviral therapy in public health facilities of Gondar City Administration, Northwest Ethiopia, based on a retrospective cohort extracted from electronic medical records, 2005‐2024 (N=12,871).

Features and categories	Values, n (%)
Age, y	
18‐27	783 (6.1)
28‐37	1741 (13.5)
38‐47	4468 (34.7)
48‐57	3998 (31.1)
58 and above	1881 (14.6)
Sex	
Female	7688 (59.7)
Male	5183 (40.3)
Marital Status	
Married	4921 (51.2)
Divorced	2243 (23.3)
Never married	1449 (15.1)
Widowed	995 (10.4)
Education level	
Higher education	1448 (15.3)
Secondary education	2953 (31.3)
Primary education	2602 (27.5)
No education	2443 (25.9)
Residence	
Urban	8878 (77.1)
Rural	2635 (22.9)
Religion	
Orthodox	8714 (91.5)
Muslim	684 (7.2)
Protestant	96 (1)
Catholic	23 (0.24)
Other	6 (0.06)

### Baseline Clinical and Immunological Characteristics

Among 12,871 people living with HIV at ART initiation, most exhibited preserved functional status (working: n=11,970, 93.1%) with low TB prevalence (n=219, 1.7% positive), consistent with late ART presentation in Ethiopian cohorts (see Table 2). The median baseline CD4 count was 225 (IQR 131‐367) cells/mm³.

**Table 2.** Baseline clinical and immunological characteristics of people living with HIV receiving antiretroviral therapy in public health facilities, Gondar City Administration, Northwest Ethiopia, 2024.

Feature and category	Values
Tuberculosis screening result, n (%)	
No	12,655 (98.3)
Yes	216 (1.7)
Functional status, n (%)	
Working	11,978 (93.1)
Ambulatory	527 (4.1)
Bedridden	366 (2.8)
Baseline CD4^a^ count (cells/mm³), median (IQR)	225 (131-367)

a CD4: cluster of differentiation 4 (a type of immune cell count).

### Balancing Dataset

**Figure 3.**
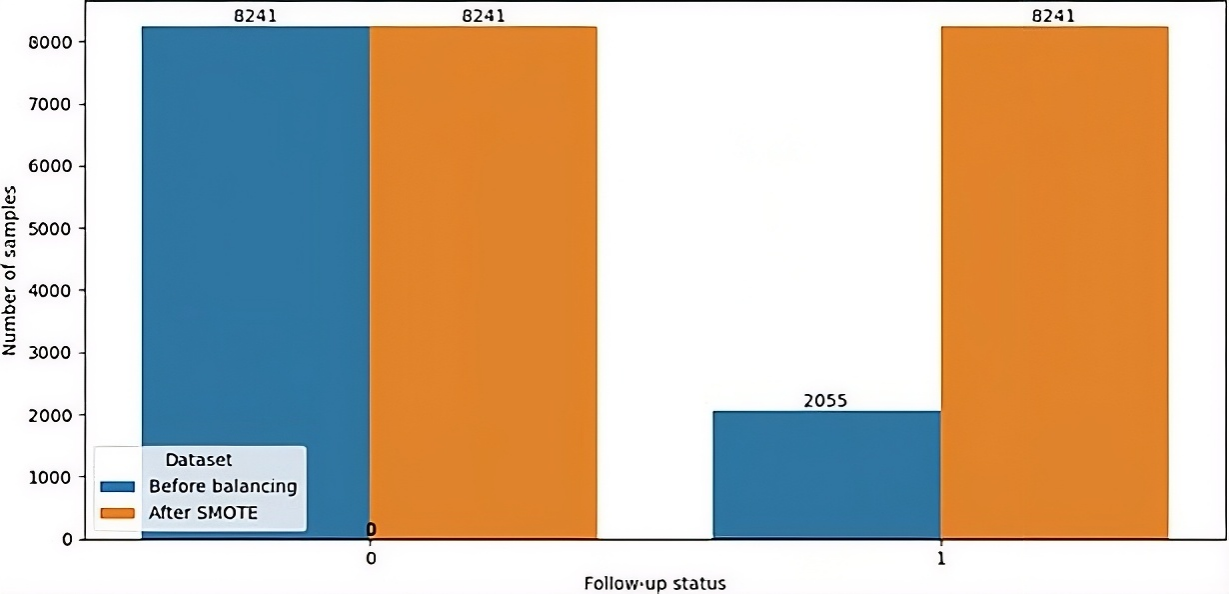
Class distribution of mortality outcomes (alive vs deceased) among adults living with HIV receiving antiretroviral therapy (ART) in Gondar City Administration, Northwest Ethiopia, showing the original imbalanced dataset and the training set after SMOTE (synthetic minority oversampling technique) was applied to address class imbalance for model development, 2005‐2024.

As seen in the descriptive statistics, the prevalence of mortality prediction among people living with HIV was 20%. This shows that the dataset was imbalanced; most observations (80%) were concentrated in the majority class (ie, alive). The SMOTE oversampling strategy added 6186 synthetic observations from the minority group (ie, dead) to balance the unbalanced distribution of the outcome variable. Therefore, the class distribution for mortality prediction among people living with HIV was balanced using SMOTE, resulting in a symmetric training dataset with 8241 observations in each class (alive and dead) to support the development of reliable and robust predictive models (see Figure 3). To avoid data leakage, the dataset was first split into training (80%) and testing (20%) subsets. SMOTE oversampling was then applied only to the training data, while the test set remained in its original, imbalanced form. This ensured unbiased performance evaluation on unseen data.

**Figure 4.**
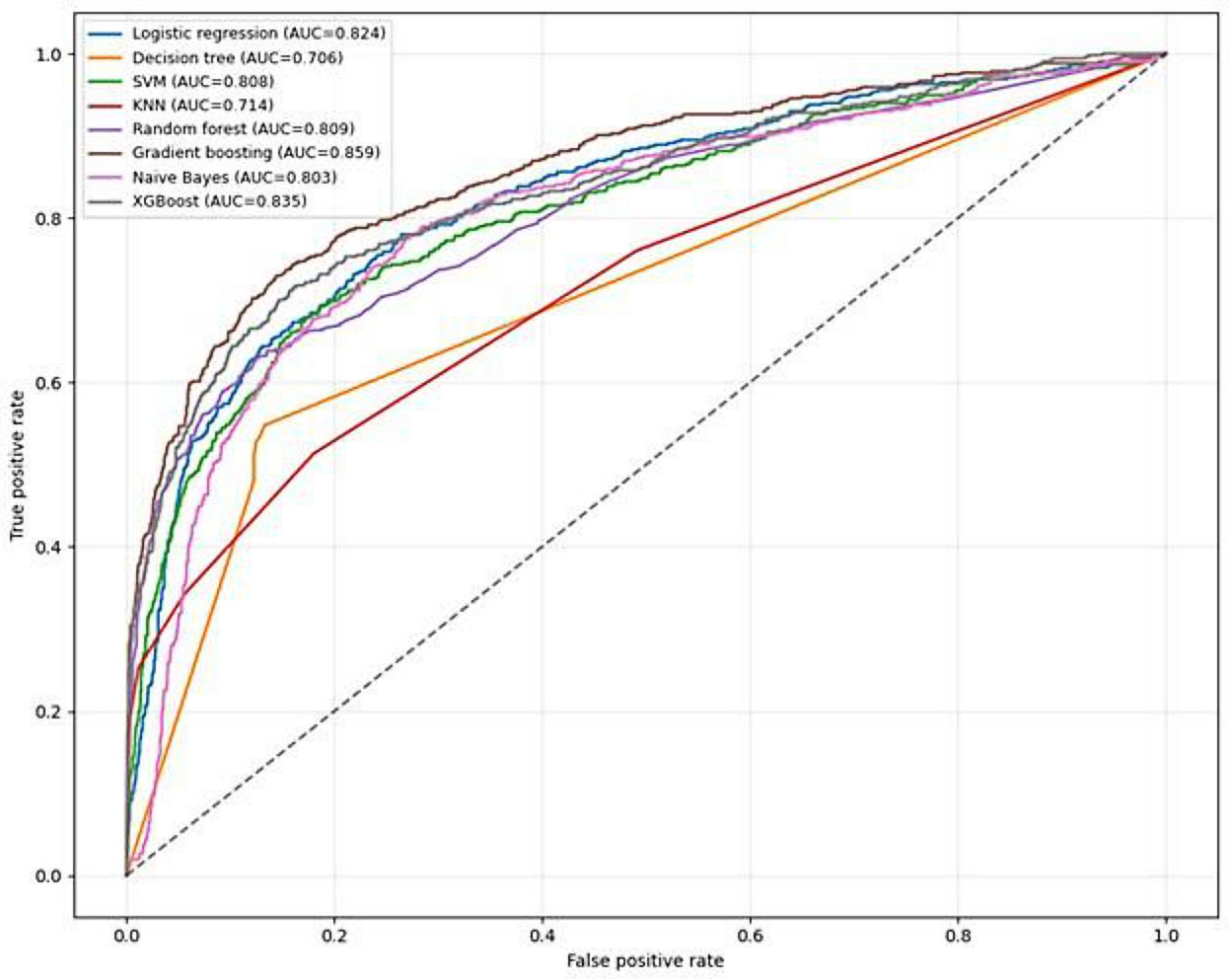
Receiver operating characteristic (ROC) curves for 7 machine-learning classifiers evaluated on the original imbalanced test dataset for mortality prediction among adults living with HIV on antiretroviral therapy (ART) in public health facilities of Gondar City Administration, Northwest Ethiopia, reporting discrimination performance before class balancing, 2005 - 2024. AUC: area under the receiver operating characteristic curve; KNN: k-nearest neighbor; SVM: support vector machine; XGBoost: extreme gradient boosting.

ROC curves for 7 ML models trained on the original imbalanced dataset (20% mortality prevalence) demonstrated clinically realistic discrimination (AUC range: 0.70‐0.86). Gradient boosting achieved superior performance (AUC=0.859), followed by XGBoost (AUC=0.835) and logistic regression (AUC=0.824). Random forest (AUC=0.809) and Naive Bayes (AUC=0.803) showed moderate discrimination, while k-nearest neighbor (KNN) and decision tree showed lower discrimination compared to ensemble models (see Figure 4).

**Figure 5.**
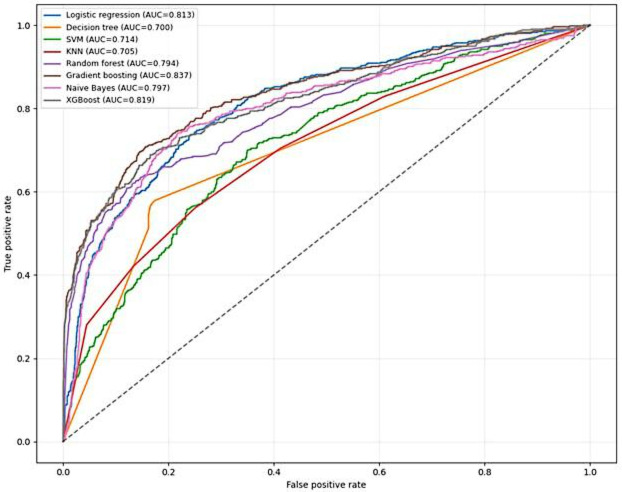
Receiver operating characteristic (ROC) curves for 7 machine-learning classifiers trained on SMOTE (synthetic minority oversampling technique)-balanced training data and evaluated on the original imbalanced test dataset for mortality prediction among adults living with HIV on antiretroviral therapy (ART) in Gondar City Administration, Northwest Ethiopia, illustrating postbalancing model discrimination, 2024. AUC: area under the receiver operating characteristic curve; KNN: k-nearest neighbor; SVM: support vector machine; XGBoost: extreme gradient boosting.

SMOTE-balanced models (training only, evaluated on the original imbalanced test set) demonstrated stable discrimination with modest recall improvements but precision trade-offs typical of oversampling. Logistic regression achieved AUC=0.813 (gain +0.0 from original), decision tree AUC=0.700 (−0.006), and KNN AUC=0.705 (−0.009). Tree-based ensembles maintained robustness: andom forest AUC=0.794 (−0.015), gradient boosting AUC=0.837 (−0.022), XGBoost AUC=0.819 (−0.016), and Naive Bayes AUC=0.797 (−0.006). Original configurations outperformed SMOTE variants (median ΔAUC=−0.013 [IQR -0.019 to 0.008]), favoring models trained on unaltered data for potential future clinical implementation, pending external validation (see Figure 5).

Model performance comparison across original and SMOTE-balanced configurations demonstrates gradient boosting original’s superiority (*F*_1_-score=0.619, AUC=0.859), outperforming XGBoost original (*F*_1_-score=0.609, AUC=0.835). SMOTE-trained models showed recall gains but precision losses: XGBoost SMOTE (*F*_1_-score=0.592, AUC=0.819; Δ*F*_1_-score=−0.017), random forest SMOTE (*F*_1_-score=0.576, AUC =0.794; Δ*F*_1_-score=−0.021), and gradient boosting SMOTE (*F*_1_-score=0.608, AUC=0.837; Δ*F*_1_-score=−0.011). Ensemble methods maintained robust discrimination after balancing (AUC>0.79), whereas simpler models showed greater variability. KNN and decision tree exhibited the lowest performance across configurations, reaffirming ensemble learning’s advantage for high-dimensional, imbalanced HIV mortality prediction.

**Figure 6.**
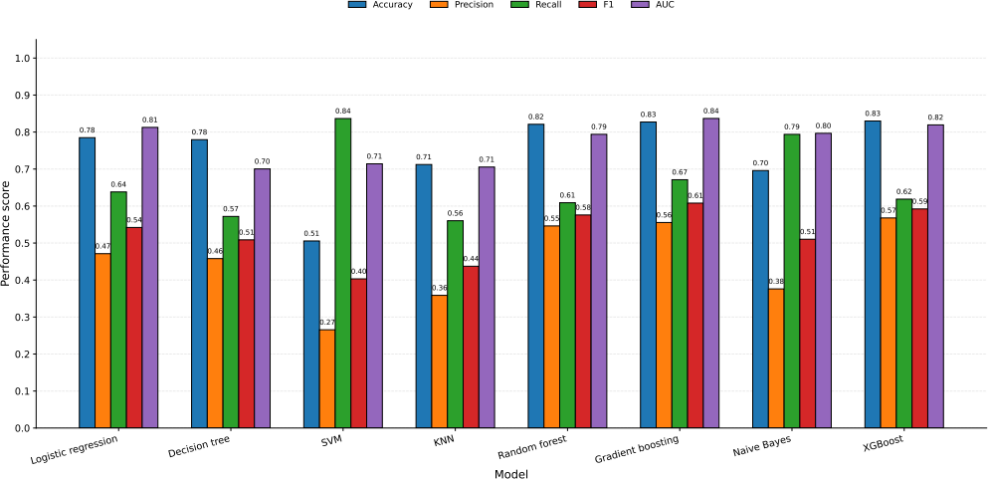
Comparative performance of machine-learning models (accuracy, precision, recall, *F*_1_-score, and area under the receiver operating characteristic curve [AUC]) for mortality prediction among adults living with HIV receiving antiretroviral therapy (ART) in public health facilities of Gondar City Administration, Northwest Ethiopia, 2024. KNN: k-nearest neighbor; SMOTE: synthetic minority oversampling technique; SVM: support vector machine; XGBoost: extreme gradient boosting.

Original data configurations consistently outperformed SMOTE variants (median Δ*F*_1_-score=−0.011, median ΔAUC=−0.013), supporting potential future clinical implementation pending external validation without synthetic oversampling. This finding aligns with real-world prevalence modeling priorities where precision preservation exceeds recall optimization for resource allocation in ART programs (see Figure 6).

### Model Building and Selection

The predictive performance of 7 ML models was comprehensively evaluated using accuracy, precision, recall, *F*_1_-score (primary), and AUC on an 80:20 stratified train-test split. A critical distinction is that all preprocessing procedures, including SMOTE, were applied to training data only; the final evaluation used the original imbalanced test set (20% mortality prevalence) to reflect real-world deployment conditions.

On the original imbalanced test data, gradient boosting demonstrated superior balanced performance (accuracy=87.0%, precision=74.5%, recall=52.9%, *F*_1_-score=0.619, AUC=0.859), outperforming XGBoost (*F*_1_-score=0.609, AUC=0.835) and random forest (*F*_1_-score=0.597, AUC=0.809). Ensemble methods consistently exceeded single classifiers, followed by Naive Bayes (*F*_1_-score=0.562) and logistic regression (*F*_1_-score=0.553).

SMOTE sensitivity analysis (training only) yielded recall gains but precision losses: XGBoost recall improved by +8.2% (*F*_1_-score=0.592, Δ*F*_1_-score=−0.017) and gradient boosting by +14.2% (*F*_1_-score=0.608, Δ*F*_1_-score=−0.011). Original configurations outperformed SMOTE variants across top models (median Δ*F*_1_-score=−0.011 [IQR 0.006-0.019], ΔAUC=−0.013 [IQR 0.008-0.019]), confirming unaltered EMR data superiority for clinical precision.

Gradient boosting (original) was selected as optimal due to the highest *F*_1_-score, cross-validation stability, and SHAP interpretability, establishing moderate-to-strong discrimination performance for 20% prevalence mortality prediction in resource-limited settings (see Table 3).

**Table 3.** Performance comparison of 7 machine-learning classifiers for binary mortality prediction among adults living with HIV on antiretroviral therapy in Gondar City Administration, Northwest Ethiopia, showing accuracy, precision, recall, *F*_1_-score, and area under the receiver operating characteristic curve (AUC) evaluated on the original imbalanced hold-out test dataset, with models trained on unbalanced and SMOTE (synthetic minority oversampling technique–balanced training data), 2024.

Model and data	Accuracy	Precision	Recall	*F*_1_-score	AUC	Δ*F*_1_-score
Gradient boosting						
Original	0.870	0.745	0.529	0.619	0.859	—^a^
SMOTE	0.827	0.556	0.671	0.608	0.837	−0.011
XGBoost^b^						
Original	0.863	0.704	0.537	0.609	0.835	—
SMOTE	0.830	0.568	0.619	0.592	0.819	−0.017
Random forest						
Original	0.855	0.672	0.537	0.597	0.809	—
SMOTE	0.821	0.546	0.609	0.576	0.794	−0.021
Logistic regression						
Original	0.852	0.696	0.459	0.553	0.824	—
SMOTE	0.785	0.471	0.638	0.542	0.813	−0.011
Naive Bayes						
Original	0.821	0.548	0.576	0.562	0.803	—
SMOTE	0.696	0.376	0.794	0.510	0.797	−0.052
Decision tree						
Original	0.806	0.513	0.527	0.520	0.706	—
SMOTE	0.779	0.458	0.572	0.509	0.700	−0.011
KNN^c^						
Original	0.824	0.605	0.342	0.437	0.714	—
SMOTE	0.712	0.359	0.560	0.437	0.705	0.000

a Not available.

b XGBoost: extreme gradient boosting.

c KNN: k-nearest neighbor.

### Association Rule Mining

Association rule mining using the Apriori algorithm (mlxtend; rules with support ≥0.065, confidence ≥0.64, and lift >4.6 were retained for interpretation) identified high-risk sociodemographic profiles among baseline characteristics. The dominant pattern—“rural residence + age 38‐47 years + no formal education + low baseline CD4 → mortality”—exhibited support=0.0686 (6.9% prevalence), confidence=68.3%, and lift=4.76, indicating that this subgroup showed a 4.76-fold higher co-occurrence with mortality compared with the baseline mortality prevalence (20% baseline prevalence).

In addition, demographic context (Christian religion, TB-negative status) maintained high lift values (4.62‐4.74), indicating that this subgroup exhibited the strongest co-occurrence pattern with mortality in the unadjusted association analysis (see Table 4). These association rules reflect co-occurrence patterns and should not be interpreted as causal risk factors.

**Figure 7.**
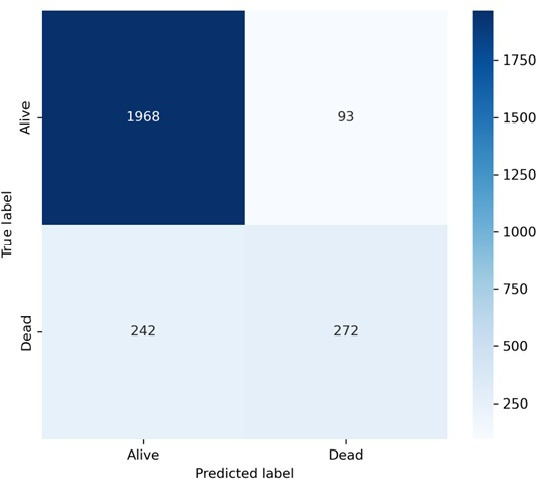
Confusion matrix for the optimized gradient boosting classifier predicting mortality among adults living with HIV receiving antiretroviral therapy (ART) in public health facilities of Gondar City Administration, Northwest Ethiopia, 2024.

The gradient boosting model demonstrates strong and clinically plausible classification performance (see Figure 7), which presents the confusion matrix of the optimized gradient boosting classifier.

**Table 4.** Association rule mining results using the Apriori algorithm showing frequent baseline sociodemographic factor combinations associated with mortality among adults living with HIV on antiretroviral therapy (ART) in Gondar City Administration, Northwest Ethiopia, 2005‐2024 (N=12,871).

Antecedent	Consequent	Support	Confidence	Lift
Rural+age 38‐47+no education+low CD4^a^	Mortality	0.0686	0.683	4.76
Christian+rural+age 38‐47+no education+low CD4	Mortality	0.0686	0.692	4.72
TB^b^-+rural+age 38‐47+no education+low CD4	Mortality	0.0651	0.680	4.74
Rural+age 38‐47+no education	Mortality	0.0707	0.668	4.65
Christian+rural+age 38‐47+no education	Mortality	0.0707	0.677	4.62
TB-+rural+age 38‐47+no education	Mortality	0.0672	0.664	4.63

a CD4: cluster of differentiation 4 (a type of immune cell count).

b TB: tuberculosis.

### Feature Ranking

**Figure 8.**
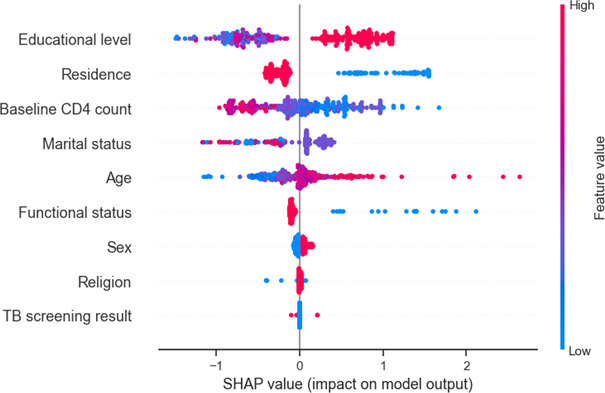
Global feature importance ranking based on mean absolute Shapley Additive Explanations (SHAP) values from the gradient boosting model, identifying the top predictors of mortality among adults living with HIV on antiretroviral therapy (ART) in Gondar City Administration, Northwest Ethiopia, 2024. CD4: cluster of differentiation 4 (a type of immune cell count); TB: tuberculosis.

Gradient boosting trained on the original dataset was selected as the optimal model, and the SHAP summary analysis was identified as the most influential baseline predictors of mortality among 12,871 people living with HIV at ART initiation. The educational level was ranked as the strongest predictor, with lower education associated with positive SHAP values (increased mortality risk) and higher education demonstrating consistent protective effects. Residence was the second most important factor, with rural residence contributing to higher predicted mortality and urban residence showing protective effects. The baseline CD4 count exhibited a clear biological gradient: lower CD4 values clustered on the positive SHAP side, indicating increased mortality risk, whereas higher CD4 counts were associated with negative SHAP values, reflecting protection. Marital status and age showed moderate influence, with older age generally increasing predicted risk. Functional status also contributed meaningfully, as ambulatory or bedridden states increased mortality risk compared to working status. In contrast, sex, religion, and TB screening results demonstrated relatively small SHAP magnitudes, indicating limited impact on overall prediction. Overall, the SHAP ranking confirms that socioeconomic vulnerability and immunological status at ART initiation are the dominant determinants of predicted mortality risk (see Figure 8).

### Waterfall Plot

The SHAP waterfall plot illustrates the individual prediction for a 45-year-old patient. The model output is expressed on the log-odds scale for the mortality class (dead=1), with the cohort baseline expected value of *E*[*f*(*X*)]=−1.886, representing the average predicted mortality risk. For this patient, the cumulative contribution of key baseline features shifts the model output to *f*(*x*)=−0.653. Because this value is substantially higher than the cohort baseline, it indicates an elevated predicted mortality risk relative to the average patient, although the absolute probability remains below 0.5.

**Figure 9.**
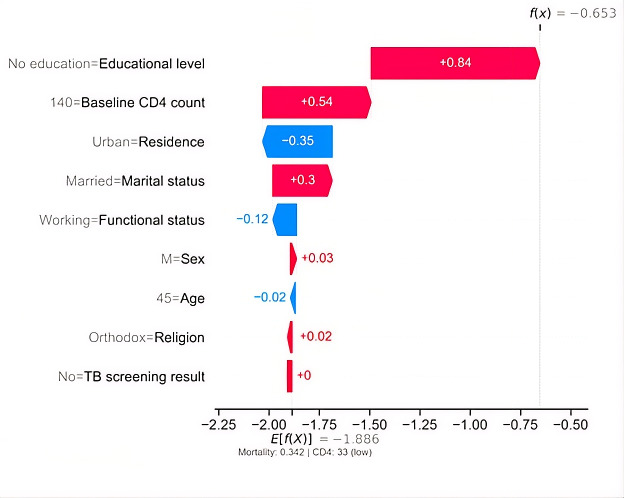
Shapley Additive Explanations (SHAP) waterfall plot illustrating an individual-level prediction from the gradient boosting mortality model for an adult living with HIV on antiretroviral therapy (ART) in Gondar City Administration, Northwest Ethiopia, 2024. CD4: cluster of differentiation 4 (a type of immune cell count); TB: tuberculosis.

Positive SHAP values (red bars) indicate features that increase predicted mortality risk, whereas negative SHAP values (blue bars) indicate protective effects. The strongest contributor to increased mortality risk was a lack of formal education (+0.84), followed by a low baseline CD4 count of 140 cells/mm³ (+0.54). Married as marital status (+0.30) and male as sex (+0.03) provided additional, smaller risk contributions. In contrast, urban residence (−0.35) and working functional status (−0.12) exerted protective effects by reducing the predicted mortality risk. Age (45 y; −0.02), religion (+0.02), and TB screening results (~0) had minimal influence on the individual prediction (see Figure 9).

## Discussion

### Principal Findings

To our knowledge, this represents one of the first Ethiopia-based applications of interpretable ensemble ML (gradient boosting combined with SHAP) using baseline EMR data to predict mortality among 12,871 people living with HIV, achieving moderate but clinically realistic performance (*F*_1_-score=0.619, AUC=0.859). Unlike earlier exploratory analyses, the final model was developed strictly using predictors recorded at ART initiation, eliminating postbaseline variables to avoid look-ahead bias and ensure valid prognostic modeling. This study contributes new evidence by demonstrating the feasibility of applying XAI models to routine EMR data to support proactive risk stratification and clinical decision-making in resource-limited settings. However, the model should be interpreted as a decision-support tool rather than a standalone clinical triage system, given its moderate sensitivity. Consistent with findings from studies published in JMIR journals, our results demonstrate the feasibility of integrating XAI models into routine clinical care to support proactive risk stratification and decision-making, particularly in low-resource settings where the efficient use of existing EMR data is critical [1-3].

Gradient boosting trained on verified baseline-only predictors achieved the best overall balance between discrimination and generalization performance (*F*_1_-score=0.619, AUC=0.859), outperforming logistic regression, Naive Bayes, KNN, and decision tree models. While XGBoost and random forest also demonstrated competitive performance, the corrected baseline-only gradient boosting model provided the most stable and clinically interpretable results for the 20% mortality prevalence setting.

SHAP values were calculated with respect to the mortality class (dead=1). Accordingly, positive SHAP values indicate an increase in predicted mortality risk, whereas negative SHAP values represent a reduction in predicted mortality risk (protective effect).

The most influential baseline predictors included educational level, baseline CD4 count, residence, marital status, and functional status. In the illustrated case, lack of formal education and a low baseline CD4 count (140 cells/mm³) exerted strong positive contributions, substantially increasing the predicted mortality risk. Urban residence and working functional status demonstrated protective contributions.

Importantly, the baseline CD4 count was modeled as a continuous predictor in the ML algorithms, and descriptive statistics have been revised to report a median of 225 (IQR 131-367) cells/mm³ to ensure methodological consistency. Unlike preliminary analyses that yielded inflated performance estimates, the corrected baseline-only model produced moderate but clinically realistic discrimination (AUC=0.859), consistent with previously published EMR-based mortality prediction studies [4-7].

### Comparison to Prior Work

These results are consistent in accuracy with studies conducted in Nigeria and Thailand, where ensemble models achieved high predictive accuracy in the clinical context [20,26]. Several studies have explored the use of ML algorithms for mortality prediction among people living with HIV. Similarly, a study from China utilizing a support vector machine reported an accuracy of 86%, which aligns with this study’s pre-SMOTE support vector machine performance [16].

The SHAP analysis provided valuable insights into the factors influencing mortality prediction.

The SHAP global importance analysis identified the educational level as the most influential baseline predictor of mortality, followed by residence, baseline CD4 count, marital status, age, and functional status. Specifically, a lack of formal education and lower baseline CD4 values were associated with an increased predicted mortality risk, whereas urban residence and working functional status were protective.

This hierarchy reflects the combined influence of socioeconomic vulnerability and biological immunosuppression at ART initiation, underscoring that both structural and clinical determinants contribute substantially to mortality risk prediction. For instance, a study conducted in Kenya found that patients on ART for extended periods had significantly lower mortality rates, reinforcing the critical role of early and consistent treatment adherence in improving survival [27].

Ensemble methods, such as XGBoost, random forest, and gradient boosting, consistently outperformed simpler models, such as logistic regression and Naive Bayes. Similar findings were reported in studies from Northern Thailand, East Africa, and Nigeria, where ensemble models achieved superior performance in health prediction tasks [28,29].

The model comparison plot demonstrated that gradient boosting achieved an accuracy of 87.0%, precision of 74.5%, recall of 52.9%, *F*_1_-score of 0.619, and AUC of 0.859. Although discrimination was strong, the recall of 52.9% indicates that nearly half of the mortality cases were not identified at the default classification threshold. Therefore, while the model demonstrates predictive capability, its sensitivity limits immediate deployment as a high-stakes early-warning system without threshold optimization.

### Limitations

This study used retrospective secondary EMR data, which may contain incomplete or inaccurately recorded variables, introducing potential information bias. Only 1 baseline record per patient was retained to avoid correlated observations, limiting longitudinal analysis. Patients who transferred to other facilities were excluded, potentially introducing selection bias.

Although the cohort required documented baseline ART initiation records, limited variable-level missingness (<10%) was addressed using median (continuous variables) and mode (categorical variables) imputation. Therefore, this was not a strict complete-case analysis. Excluding individuals on ART for less than 6 months may have introduced survivorship bias, potentially inflating performance metrics and limiting generalizability to newly initiated ART populations.

SMOTE-based oversampling may not fully replicate real-world population distributions. External validation using independent datasets was not performed, and model performance may vary in other geographic or clinical contexts. Data were drawn from a single city administration, limiting external validity.

Finally, given the moderate sensitivity (52.9%, 272/514), the model may miss a substantial proportion of high-risk patients at the default threshold. Future implementations should consider threshold calibration based on clinical priorities, particularly if minimizing false negatives is paramount.

### Future Directions

Future studies should use multicenter or national-level data to enhance generalizability and external validity. Prospective validation studies are needed to evaluate real-world performance. Threshold optimization strategies should be explored to improve sensitivity where early mortality detection is prioritized. Integrating temporal modeling approaches may capture longitudinal treatment dynamics. Additionally, embedding explainable models within clinical workflows could support structured risk assessment, provided that implementation is accompanied by clinician oversight and contextual calibration.

### Conclusion

This study demonstrates that interpretable ensemble ML models trained exclusively on baseline EMR data achieved moderate yet clinically meaningful discrimination in predicting mortality risk among people living with HIV initiating ART. SHAP-based interpretation confirmed that educational attainment and baseline CD4 count were the most influential predictors, followed by residence, age, and functional status, highlighting the combined impact of socioeconomic vulnerability and immunological status at treatment initiation. These findings suggest that transparent and explainable ML approaches may support early risk stratification and targeted intervention planning within HIV care programs. Nevertheless, external validation and prospective evaluation are required before integration into routine clinical decision-making.

## Abbreviations

ART: antiretroviral therapy
AUC: area under the receiver operating characteristic curve
CD4: cluster of differentiation 4 (a type of immune cell count)
EMR: electronic medical record
KNN: k-nearest neighbor
ML: machine learning
ROC: receiver operating characteristic
SHAP: Shapley Additive Explanations
SMOTE: synthetic minority oversampling technique
TB: tuberculosis
UNAIDS: Joint United Nations Program on HIV/AIDS
XAI: explainable artificial intelligence
XGBoost: extreme gradient boosting
